# The effect of bilateral cardiac sympathetic denervation for refractory ventricular tachycardia in ischemic cardiomyopathy

**DOI:** 10.1002/joa3.12336

**Published:** 2020-03-27

**Authors:** Yuko Miki, Takehito Sasaki, Yoshinori Okazaki, Mitsuho Inoue, Katsura Niijima, Hiroyuki Motoda, Yutaka Take, Kentaro Minami, Nogiku Niwamae, Mitsuhiro Kamiyoshihara, Kohki Nakamura, Shigeto Naito

**Affiliations:** ^1^ Division of Cardiology Gunma Prefectural Cardiovascular Center Maebashi Gunma Japan; ^2^ Department of Cardiovascular Medicine Maebashi Red Cross Hospital Maebashi Gunma Japan; ^3^ Department of General Thoracic Surgery Maebashi Red Cross Hospital Maebashi Gunma Japan

**Keywords:** cardiac sympathetic denervation, ischemic cardiomyopathy, sympathetic nervous system, ventricular tachycardia

## Abstract

Recent studies have shown that cardiac sympathetic denervation (CSD) is effective in the treatment of refractory ventricular tachyarrhythmia in patients with structural heart disease. This case report aimed to highlight the effect of bilateral CSD in suppressing treatment‐resistant ventricular tachycardia in patients with ischemic cardiomyopathy.

## Graphical abstract

We present our experience with bilateral cardiac sympathetic denervation (CSD) in a patient with a history of implantable cardioverter defibrillator implantation and ischemic cardiomyopathy who suffered treatment‐resistant ventricular tachycardia (VT) storm, including implantable cardioverter defibrillator‐reprogramming, antiarrhythmic drug, and catheter ablation except sedation. After bilateral CSD through video‐assisted thoracoscopic approach, this refractory VT was completely suppressed, and no recurrence was observed at the 1‐month follow‐up.

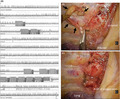

## 1 INTRODUCTION

The sympathetic nervous system plays an important role in life‐threatening ventricular arrhythmia, especially for electrical ventricular tachycardia (VT) storm in structural heart disease (SHD). The antiadrenergic interventions are considered a therapeutic strategy for SHD patients with refractory ventricular arrhythmia.

## 2 CASE REPORT

**FIGURE 1:**
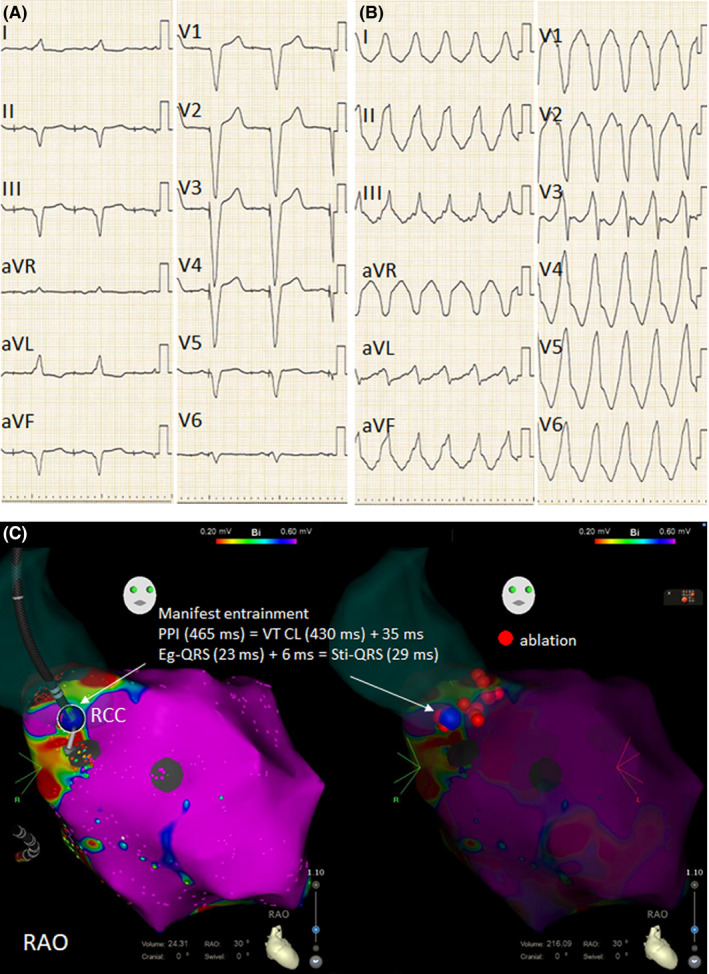
A, 12‐lead ECG showed A paced, V paced. B, 12‐lead ECG showed clinical VT. VT CL was 430 ms C, RAO view of substrate mapping with voltage criteria of 0.2‐0.6 mV for the LVA. Substrate mapping during right ventricular pacing with a CARTO system was performed using a multielectrode catheter. LVA was observed on the inferior and lateral LV wall. Perfect pace‐mapping was obtained at ostium of the RCA. Electrophysiological findings suggested that the ventricular myocardium captured by RCC was close to the exit site of the reentrant circuit of VT. RF energy was applied for RCC and left ventricle on the opposite side of the RCC and abandoned it for the ostium of the RCA where the perfect match of pace‐mapping was obtained. blue circle, site of pace‐mapping at RCC; CL, cycle length; Eg‐QRS, electrogram‐QRS intervals; PPI, postpacing interval; RAO, right anterior oblique; RCA, right coronary artery; RCC, right coronary cusp; red circle, ablation site; RF, radiofrequency; Sti‐QRS, stimulus‐QRS intervals; VT, ventricular tachycardia

**FIGURE 2:**
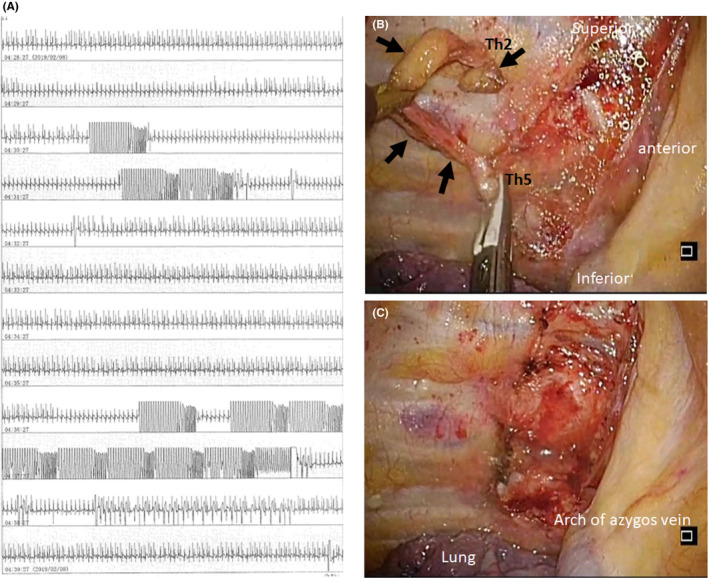
A, Two days after ablation, electrical VT storm recurred following cessation of sedation. Continuous telemetry rhythm strip showed 11 antitachycardia pacing therapies and 1 defibrillation within 8 min. B, Videoscopic still frame of VATS‐right CSD demonstrated the sympathetic chain from the second to the fifth thoracic ganglion before resection. C, The right sympathetic chain from the second to the fifth thoracic ganglion was resected. CSD, cardiac sympathetic denervation; VATS, video‐assisted thoracoscopic surgery; VT, ventricular tachycardia

A 79‐year‐old man with a history of implantable cardioverter defibrillator (ICD) implantation and ischemic cardiomyopathy presented with ICD shock associated with a feeling of faintness. He received an appropriate ICD shock and antitachycardia pacing therapy for VT at 148 beats per minute. He had undergone conservative medical treatment for myocardial infarction 30 years previously. He had received ICD implantation for secondary prevention of sustained VT 3 years previously. At the same time, coronary angiogram revealed chronic total occlusion of the right coronary artery (RCA) at the proximal portion with well‐developed collateral artery from the left circumflex artery to the distal RCA. The proximal left anterior descending artery had 75% stenosis. The obtuse marginal branch was occluded. He did not undergo coronary revascularization because adenosine myocardial perfusion imaging showed perfusion defect during stress in the inferior and inferolateral region, consistent with the RCA territory and did not reveal evidence of reversible ischemia. His medical history was significant for cerebral infarction, diabetes mellitus, and hypothyroidism. His current medication with respect to antiarrhythmic drugs included amiodarone hydrochloride 100 mg and bisoprolol fumarate 1.25 mg. Transthoracic echocardiography showed akinesis of the inferior and inferolateral left ventricular (LV) wall and dyskinesis of the apex. The left ventricle was enlarged and LV wall motion was reduced (ejection fraction 27%). On day 11 of admission, he experienced VT storm and received an appropriate ICD shock and two sessions of antitachycardia pacing therapy despite escalating doses of bisoprolol from 1.25 to 3.75 mg per day. Twelve‐lead ECG showed that the QRS morphology during clinical VT was identical with that which occurred 3 years previously. In addition, he was treated with landiolol and escalating doses of amiodarone hydrochloride from 100 to 150 mg per day. However, on day 16 of admission, he experienced VT storm again and received multiple ICD shocks and antitachycardia pacing therapies. This VT was refractory to antiarrhythmic drugs, including amiodarone, bisoprolol, and intravenous landiolol and suppressed via sedation with propofol. On day 18 of admission, electrophysiological study and catheter ablation were performed. LV endocardial voltage mapping with a 3D electroanatomical system was performed during right ventricular pacing using a multielectrode catheter. Low‐voltage area (LVA) was observed on the inferior and lateral LV wall. Perfect pace‐mapping was obtained at the ostium of the RCA. Pace‐mapping at the right coronary cusp (RCC) was identical to clinically documented VT and was slightly inferior compared with that at the ostium of RCA. Stimulus‐QRS interval at RCC was 29 ms Then, clinical VT was induced with right ventricular rapid pacing. The cycle length (CL) of clinical VT was 430 ms An entrainment mapping at the RCC showed a manifest entrainment; however, the QRS morphology was similar to the QRS morphology of clinical VT, electrogram‐QRS intervals (23 ms) matched the stimulus‐QRS intervals (29 ms), and the postpacing interval was 465 ms These findings suggested that the ventricular myocardium captured by RCC was close to the exit site of the reentrant circuit of VT. We performed radiofrequency (RF) applications for RCC and left ventricle on the opposite side of RCC and abandoned it for the ostium of the RCA where the perfect match of pace‐mapping was obtained. We finished this session without confirming the inducibility of VT because he developed cardiogenic shock and required intra‐aortic balloon pumping support. Two days after ablation, electrical VT storm recurred following cessation of sedation. In this case, VT was refractory to the multimodality approach, including ICD‐reprogramming, antiarrhythmic drug, and catheter ablation except sedation. On day 27 of admission, bilateral cardiac sympathetic denervation (CSD) (Th2‐5) through video‐assisted thoracoscopic approach was performed. After bilateral CSD, this refractory VT was completely suppressed, and no recurrence was observed at the 1‐month follow‐up. However, he had developed acute myocardial infarction (AMI) on day 52 of admission and died on day 54 (Figures 1 and 2).

## 3 DISCUSSION

The efficacy of CSD for refractory VT is well known. The pharmacologic and surgical antiadrenergic interventions, a β‐adrenergic blocking agent and a left CSD reduce sudden cardiac death in postmyocardial infarction patients.^1^ Recent studies have reported more beneficial effects of bilateral CSD compared to left CSD.^2^, ^3^ In this case, we could not demonstrate sympathetic hyperactivity via increased LF/HF because he had pacing dependency. Clinical VT occurred in an awake state and never happened while he was asleep or sedated,therefore, CSD was attempted. Although this refractory VT was completely suppressed after bilateral CSD, he died because of AMI on postoperative day 27. Vaseghi et al reported the outcomes of 121 patients with SHD (ejection fraction of 30% ± 13%) who underwent CSD for refractory VT or ventricular fibrillation storm.^2^ CSD reduced VT/ICD shocks; however, 31 of 121 patients had died, and 10 had undergone orthotopic heart transplantation after a median follow‐up of 1.1 years (IQR: 0.4‐2.4). Multivariable analyses showed that preprocedure advanced NYHA, longer VT CL, and a left‐sided only procedure were independent variables associated with recurrent ICD shocks, transplant, or death. This case highlights the effect of bilateral CSD for refractory VT in patients with ischemic cardiomyopathy, considering that patient prognosis was not good.

## CONFLICT OF INTEREST

The authors declare no conflict of interest for this article.
